# Sarcoidosis presenting as hiccups

**DOI:** 10.1002/rcr2.605

**Published:** 2020-06-29

**Authors:** Melissa Neumann, Kevin G. Lazo, Diane Stover

**Affiliations:** ^1^ Department of Medicine Northwell Health Manhasset NY USA; ^2^ Division of Pulmonary Medicine Memorial Sloan Kettering Cancer Center New York NY USA

**Keywords:** Chronic hiccups, hiccups, persistent hiccups, sarcoidosis

## Abstract

Hiccups are common; however, hiccups caused by sarcoidosis have rarely been reported. An unusual case involving a patient with persistent hiccups possibly caused by hilar/mediastinal lymph node enlargement due to sarcoidosis prompted us to perform a literature search. Eight case reports relating hiccups to sarcoidosis were found and in only one case were the hiccups thought to be due to thoracic lymphadenopathy (LAD). Most cases were attributed to involvement of the central nervous system (CNS) with sarcoidosis. Management of hiccups in general is unclear and only chlorpromazine is approved by the Food and Drug Administration (FDA) for treatment; multiple other pharmacological agents have been advocated mostly being ineffective. This case report describes a patient whose hiccups were likely caused by thoracic sarcoidosis. It reviews the mechanisms of hiccups, explores co‐morbid conditions associated with hiccups (including sarcoidosis), and provides some recommended treatments.

## Graphical abstract

Hiccups caused by sarcoidosis have rarely been reported. We present an unusual case of sarcoidosis presenting with persistent hiccups that we believe is caused by his hilar and mediastinal lymph node enlargement.

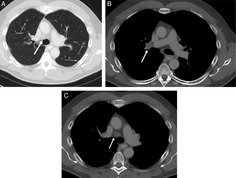

## Introduction

Sarcoidosis is a chronic granulomatous disease most common in young adults and especially African Americans. According to the U.S. census data, there are over 25,000 new cases of sarcoidosis diagnosed in the United States annually, with 185,000 patients seeking medical care. Most patients have pulmonary involvement, although many other organs can be affected such as the heart, skin, eyes, and central and peripheral nerves. Here, we present an unusual case of sarcoidosis presenting with persistent hiccups that we believe is caused by his hilar and mediastinal lymph node enlargement.

## Case Report

A 64‐year‐old male was referred to the pulmonary clinic because of an abnormal radiograph found during an evaluation for persistent hiccups. The hiccups developed after a procedure to excise a cyst on his left thumb. To determine the hiccup aetiology, the patient had a computed tomography (CT) scan of his chest, abdomen, and pelvis. He was found to have mildly enlarged mediastinal/hilar lymph nodes and pulmonary nodules as well as a left renal mass. Other than hiccups, the patient had no cough, sputum production, shortness of breath, chest pain, and no constitutional symptoms such as fever, night sweats, decreased appetite, or weight loss. Past medical history and review of systems were unremarkable.

Physical examination demonstrated a healthy appearing male in no acute distress; his vital signs were normal; the skin had a large keloid on the anterior chest wall (long‐standing from an ingrown hair that was excised), no rashes, and no lacrimal gland enlargement; the lungs were clear both to auscultation and percussion; the heart showed a regular rhythm with no murmurs or gallops; the abdomen was soft and non‐tender, no masses or organomegaly were palpated; the extremities were without clubbing, cyanosis, or oedema; neurological examination showed no cranial nerve abnormalities with sensation and reflexes intact and no muscle weakness; and there was no supraclavicular, cervical, or axillary lymphadenopathy (LAD).

**Figure 1:**
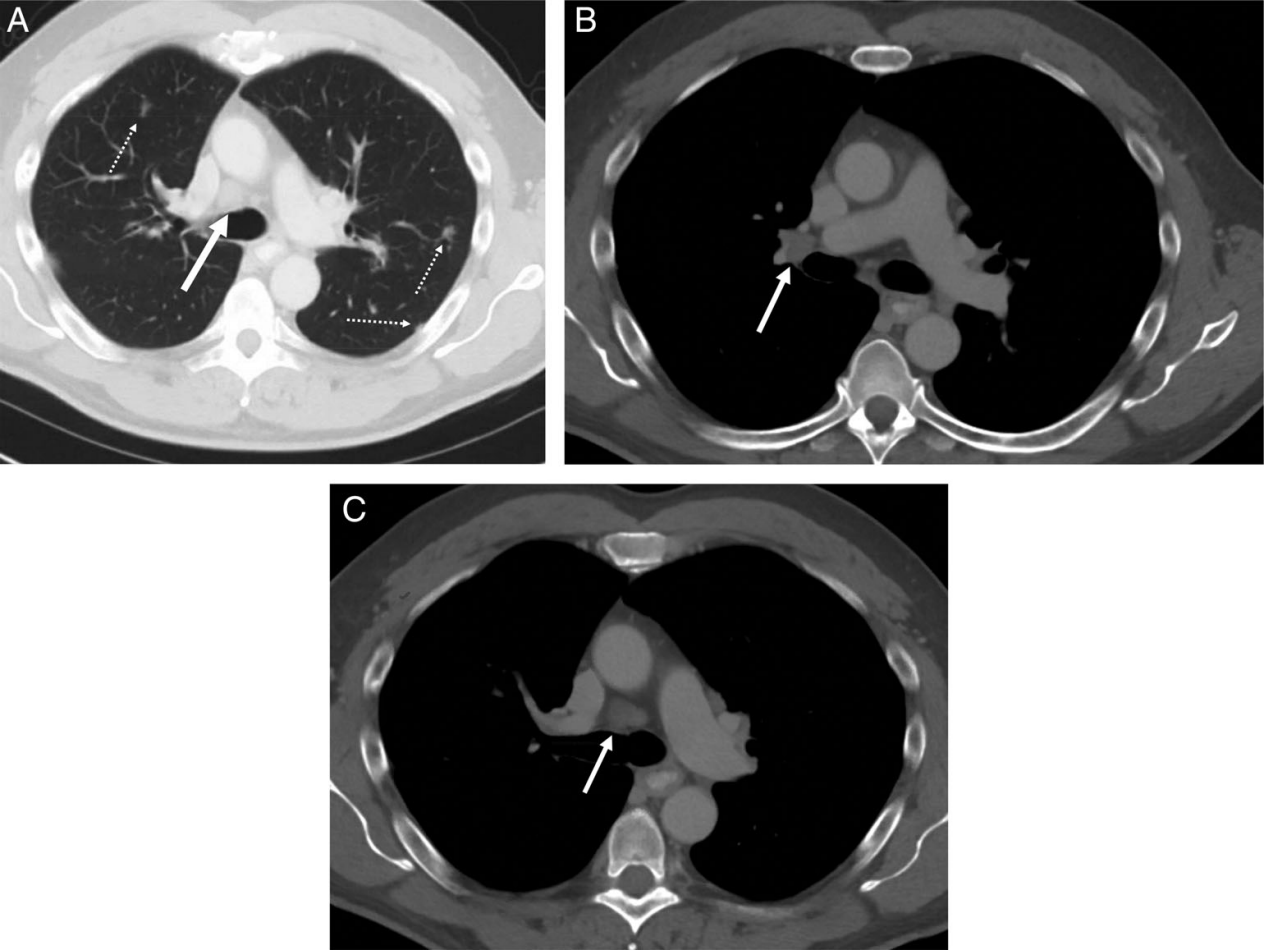
(A) A computed tomography (CT) scan of the patient's chest in lung window. Note the white dashed arrows point to pulmonary nodules found on this patient. The large white arrow shows the right pre‐tracheal lymphadenopathy (LAD). (B) A CT scan of the patient's chest in the mediastinal window, showing the right hilar LAD that measures 10.88 mm × 16.55 mm. (C) A CT scan of the patient's chest in the mediastinal window, showing the right pre‐tracheal LAD that measures 19.01 mm × 15.35 mm.

Chest CT scan showed fibronodular changes in the lung apices, likely reflecting prior granulomatous disease, also seen were scattered pulmonary nodules and hilar LAD and mild right pre‐tracheal LAD (Fig. 1). A positron emission tomography (PET) scan demonstrated a minimally hypermetabolic left kidney lesion suspicious for renal cell carcinoma and hypermetabolic bilateral lung nodules, hilar lymph nodes, and bilateral axillary nodes. Laboratory data revealed a normal metabolic profile, complete blood count, and angiotensin‐converting enzyme level.

A pathological analysis of the pulmonary nodules and enlarged mediastinal lymph nodes was sought to make sure the patient did have not have metastatic renal cancer.

The hiccups persisted despite multiple therapies, including proton pump inhibitors, a course of prednisone, and a variety of muscle relaxants. He first underwent endobronchial ultrasound without a definitive diagnosis. He then had a mediastinoscopy with removal of the pre‐tracheal lymph nodes, which made the diagnosis of sarcoidosis.

Interestingly, after the mediastinoscopy, the patient's hiccups resolved. No further treatment was necessary, and the patient remained without further episodes of hiccups. The patient underwent a nephrectomy to remove the renal mass, which was a renal cell carcinoma. He is presently without any evidence of cancer or active disease with sarcoidosis, and he remains without further episodes of hiccups.

## Discussion

A hiccup is defined as an involuntary contraction of the diaphragm causing inspiration, followed by closure of the glottis resulting in the “hic”cup sound, a protective reflex to prevent significant hyperventilation.

Hiccups are commonly associated with abnormalities involving the neurological, gastrointestinal, and respiratory systems. Its mechanism involves the central and peripheral nerves, both afferent and efferent signalling. The reflex arc involves the afferent limb which includes the glossopharyngeal, vagus, and phrenic nerves, and sympathetic chain. Central mediators for hiccups are not well understood but are believed to involve the midbrain, including the medulla oblongata, periaqueductal grey matter, subthalamic nuclei, phrenic nerve nuclei, reticular formation, and hypothalamus. The hiccup centre is thought to be located in the midbrain (medulla) or high cervical spinal cord between C3 and C5. The efferent limb involves the nerves stimulating the glottis, the phrenic nerve, and inspiratory intercostal muscles. Although there is a lack of studies, hiccups generally result from lesions within the hiccup reflex limb [10, 11, 12].

Persistent hiccups are defined as lasting for more than 48 h to one month, and chronic lasting for more than one month. The Guinness medical record breakers state that the longest attack of hiccups was by Charles Osborne (1894–1991) of Anthon, Iowa. He started hiccupping in 1922 while attempting to weigh a hog before slaughtering it. Physicians were unable to find a cure and he continued hiccupping until February 1990, for a total of 68 years.

Serious health complications of hiccups involve malnutrition, weight loss, dehydration, insomnia, and even death in extreme situations. It is estimated that 4000 hospital admissions are due to hiccups annually. A report of 18 cases of persistent hiccups demonstrated the following co‐morbid conditions: vascular lesions (20%), post‐operative causes (18%), central nervous system (CNS) disease (17%), and duodenal ulcer (5%). Central causes of hiccups include various types of strokes such as lateral medullary infarcts, and other injuries to the CNS. Peripheral causes include mediastinal tumours, myocardial ischaemia, gastroesophageal reflux diseaseand chest trauma [10, 11]. Interestingly, in a study involving 220 cases of intractable hiccups, while 17% had CNS lesions (most often strokes) only one was attributed to sarcoidosis.

There have been eight case reports prior to ours demonstrating a link between sarcoidosis and hiccups (Table 1). Five of the patients had proven CNS sarcoidosis. Four were successfully treated with corticosteroids and one patient had a poor response to treatment, resulting in death. Among the five cases with hiccups thought to be due to CNS involvement, all [1, 3, 5, 7, 8] showed neurological abnormalities, elevated protein and/or cell count within the cerebrospinal fluid, and all had abnormal brain imaging. Kondo et al.'s case, reporting hiccups caused by CNS sarcoid, is perplexing as there were no neurological symptoms reported and the CT scan and magnetic resonance imaging (MRI) of the brain were normal [2]. Hackworth et al. reported a patient with peritoneal lesions and hiccups with a poor response to multiple therapies [4]. The authors attributed the cause of hiccups to peritoneal lesions without an explanation of its mechanism. Both patients had mediastinal LAD similar to our case, which may have been the culprit.

**Table 1:** Characteristics of sarcoid patients with persistent hiccups.

Case report	Age, gender	Symptoms and/or physical examination	Imaging	Proposed cause of hiccups	Response to steroids
Douglas et al., 1973 [1]	38, M	Aphasia, headaches, grand mal seizures, deafness	chest x‐ray: bilateral hilar adenopathy	CNS sarcoidosis (hilar adenopathy had resolved and hiccups persisted)	Poor
		Erythema nodosum, hepatosplenomegaly, optic atrophy bilaterally	Pneumoencephalography: dilated lateral ventricles, non‐filling left temporal horn (nodules in leptomeninges on craniotomy)		
Kondo et al., 1989 [2]	67, M	Crepitant rales over left lateral thorax and 8 mm left supraclavicular lymph node	Chest CT: mediastinal adenopathy	Reported as CNS sarcoidosis but possibly due to mediastinal LAD	Yes
			Brain CT and MRI: normal		
Connolly et al., 1991 [3]	25, M	Urinary retention. Uveitis, peripheral facial nerve palsy, ataxic gait, bilateral upper and lower extremity weakness	chest x‐ray: bilateral hilar adenopathy	CNS sarcoidosis (due to significantly abnormal brain and spinal MRI)	Yes
			Brain and spinal MRI: nodular thickening of the leptomeninges, enlarged fifth cranial nerve		
Hackworth et al., 2009 [4]	61, M	Vomiting triggered by hiccups, 50 lb weight loss, erosive oesophagitis, ascites without hepatomegaly	Chest CT: mediastinal adenopathy with scattered pulmonary, hepatic, and splenic lesions consistent with sarcoidosis	Reported as peritoneal irritation but cause may be mediastinal LAD	Poor
			Brain MRI: normal		
Miura et al., 2010 [5]	56, M	Urinary incontinence and constipation	Chest CT: post‐inflammatory changes in upper and middle lung fields with slight mediastinal adenopathy	CNS sarcoidosis (likely due to significantly abnormal brain MRI)	Yes (in combination with clonazepam)
		Bradykinesia, mild rigidity, spasticity, mask‐like face myoclonus of right lower limb, hyporeflexia, postural instability, right leg myoclonus	Brain MRI: changes in the periventricular and deep white matter bilaterally		
Lin et al., 2010 [6]	48, M	Normal physical examination	Chest CT: mediastinal adenopathy	Pulmonary sarcoidosis with thoracic LAD	No (given and unable to tolerate)
Seby et al., 2012 [7]	45, M	Right‐sided Horner's syndrome, diffuse hyperreflexia, hoarseness, dysphagia, right‐sided vocal cord paralysis with hypophonic speech	Neck and chest CT: lateral deviation of the right true vocal cord with calcified mediastinal and hilar adenopathy	CNS sarcoidosis (due to abnormal brain MRI)	Yes (in combination with methotrexate)
			Brain MRI: focused hyperintensity in right dorsomedial medulla involving dorsal motor nucleus and solitary nucleus		
Chen et al., 2018 [8]	55, F	Progressive numbness and sensory disturbance of distal extremities	Chest CT: bilateral supraclavicular, hilar, mediastinal adenopathy	CNS sarcoidosis (due to abnormal brain MRI)	Yes
			Brain MRI: circumscribed mass lesion on the medulla oblongata		

CNS, central nervous system; CT, computed tomography; CXR, chest x‐ray; F, female; LAD, lymphadenopathy; M, male; MRI, magnetic resonance imaging.

Another case (Lin et al.) was a patient without CNS involvement but who had right hilar LAD and multiple mediastinal lymph node involvement on chest CT scan [6]. Hiccups were attributed to the enlarged mediastinal lymph nodes compressing the phrenic nerve (Table 1). This case is the only one we could find which attributed the hiccups to thoracic LAD caused by sarcoidosis. In this patient, the enlarged lymph nodes were located both to the right of the trachea and posteriorly to the ascending aorta (Fig. 1B, C). The phrenic nerve runs to the right and anterior to the trachea. Both the left sympathetic chain and the inferior cardiac branch of the vagus nerve run within the cardiac plexus, located posterior to the heart and anterior to the trachea. We propose that this patient's hiccups were caused by either innervation of the right phrenic nerve, the cervical cardiac branch of the sympathetic trunk, or the vagus nerve.

Our case report highlights a patient with persistent hiccups likely due to enlarged mediastinal lymph nodes; with removal of the mediastinal lymph nodes after mediastinoscopy, the hiccups subsided.

The response to steroids in patients with hiccups due to sarcoidosis has been variable. In the small number of cases reported with predominantly CNS sarcoidosis, more than half responded to steroids alone or in combination with another drug [9]. Alternative or combination treatments for hiccups in general include chlorpromazine, other dopamine agonists (such as haloperidol and metoclopramide), gabapentin, serotonin, and histamine agonists. Non‐pharmacological treatments that have been advocated include phrenic nerve block, vagus nerve stimulator, acupuncture, and breathe holding.

The only case in the literature that attributed hiccups to sarcoid LAD (Lin et al.) was unable to tolerate steroids [6]. Kondo et al. found that steroids decreased the hiccups, but when tapered the hiccups recurred [2]. Our patient had no response to corticosteroids and a multiplicity of other drugs including benzodiazepines, proton pump inhibitors, and muscle relaxants, all of which have been used for intractable hiccups with variable success. Although our patient's hiccups were refractory to medical management, his hiccups resolved after surgical removal of the mediastinal lymph nodes.

### Disclosure Statement

Appropriate written informed consent was obtained for publication of this case report and accompanying images.
